# Is there collaboration specific neurophysiological activation during collaborative task activity? An analysis of brain responses using electroencephalography and hyperscanning

**DOI:** 10.1002/brb3.2270

**Published:** 2021-10-07

**Authors:** Paul Léné, Alexander J. Karran, Elise Labonté‐Lemoyne, Sylvain Sénécal, Marc Fredette, Kevin J. Johnson, Pierre‐Majorique Léger

**Affiliations:** ^1^ Département de management HEC Montréal Montréal Quebec Canada; ^2^ Département de technologies de l'information HEC Montréal Montréal Quebec Canada

**Keywords:** collaboration neuroscience, EEG, electroencephalography, hyperscanning, social neuroscience

## Abstract

Collaboration between two individuals is thought to be associated with the synchrony of two different brain activities. Indeed, prefrontal cortical activation and alpha frequency band modulation has been widely reported, but little is known about interbrain synchrony (IBS) changes occurring during social interaction such as collaboration or competition. In this study, we assess the dynamic of IBS variation in order to provide novel insights into the frequency band modulation underlying collaboration. To address this question, we used electroencephalography (EEG) to simultaneously record the brain activity of two individuals playing a computer‐based game facing four different conditions: collaboration, competition, single participation, and passive observation. The computer‐based game consisted of a fast button response task. Using data recorded in sensor space, we calculated an IBS value for each frequency band using both wavelet coherence transform and phase‐locking value and performed single‐subject analysis to compare each condition. We found significant IBS in frontal electrodes only present during collaboration associated with alpha frequency band modulation. In addition, we observed significant IBS in the theta frequency band for both collaboration and competition conditions, along with a significant single‐subject cortical activity. Competition is distinguishable through single‐subject activity in several regions and frequency bands of the brain. Performance is correlated with single‐subject frontal activation during collaboration in the alpha and beta frequency band.

## 1 INTRODUCTION

Driven by technological progress and insights provided by social neuroscience concerning the interactive nature of interpersonal and team cognition, understanding brain activity dynamics when performing collaborative or competitive tasks has gained importance and popularity (Babiloni & Astolfi, 2014). However, it remains unclear whether collaboration or competition is associated with specific brain networks and frequency band modulation. Therefore, refining and increasing our understanding of the underlying brain dynamics and mechanisms of social interactions during task activities will contribute to the further development of the field and related applications.

Collaboration is perhaps the most studied social interaction in social neuroscience (Babiloni & Astolfi, 2014). Collaboration between humans is defined as the act of working together to achieve either multiple or singular goals, where the success of an individual depends on the success of another person with which the first individual is interacting in order to achieve that common goal (Mead, 2018; Wood & Gray, 1991). In contrast, competition between humans is defined as the act of seeking or striving to gain, what another or others are endeavoring to gain at the same time (Mead, 2018). From a neurophysiological perspective, these two interactions represent specific cases within the field of social neuroscience, when considering that interactions related to either collaboration or competition are shaped by the dynamics of any actions undertaken to achieve a particular goal (e.g., playing music vs. problem‐solving).

Collaboration and competition also echo an important conceptual model in social neuroscience, referred to as the theory of mind (ToM; Gallagher & Frith, 2003). ToM outlines a conceptual framework that provides a basis for assessing an individual's capacity for empathy and understanding of others by partitioning human social cognition into “self” and “other.” Within this framework, the investigation, explanation, and prediction of an individual's behavior within a social grouping are enabled by attributing independent mental states, such as beliefs, desires, emotions, or intentions and proposing which states may reach consonance during shared activities.

More precisely, inter‐human collaboration has been associated with specific brain networks. Indeed, research results from studies involving collaborative tasks appear to converge toward an increase of interbrain synchrony (IBS) in the frontal (Astolfi, Toppi, et al., 2010; Babiloni et al., 2012; Dumas et al., 2010; Konvalinka et al. et al., 2014; Wilson et al., 2008; Yun et al., 2008) and prefrontal areas (Astolfi et al., 2010; Babiloni et al., 2006; Cui et al., 2012; Funane et al., 2011; Labonte‐Lemoyne et al., 2016; Lindenberger et al. et al., 2009; Schippers et al., 2010). These studies indicate that the modulation of multiple frequency bands may play a crucial role in the collaboration process. Indeed, there are indications that these modulations are expressed as an increase within the alpha, beta, and theta frequency bands depending on task type (Babiloni et al., 2012; Dikker et al., 2017; Dumas et al., 2010; Labonte‐Lemoyne et al., 2016; Pérez et al., 2017; Sinha et al., 2017; Toppi et al., 2016).

From a technical and technological perspective, research within social neuroscience and IBS has opened a window to multiple discoveries and clinical applications. Indeed, meta‐analysis and research findings have deepened our understanding of the neural structures and cognitive processes contributing to social behaviors through the development of new experimental methods, sensor technologies, and tools for data analysis (Babiloni & Astolfi, 2014; Hagan et al., 2008; Phan et al., 2002). Furthermore, these new methods and findings have been amalgamated into clinical practice contributing to the quality of life improvements among patients presenting social deficits (Astolfi, Toppi, et al., 2012; Babiloni & Astolfi, 2014; Chiu et al., 2008; Piggot et al., 2004; Reiss, 2004; Watson et al., 2008). In addition, social neuroscience has provided contributions to the direction of human cognitive augmentation in human‐human, human‐machine teaming, providing insight to improve team cooperation optimization (Schmorrow & Fidopiastis, 2011; Stevens et al., 2013). Finally, when viewed as a whole, the field now offers alternative research paradigms and additional layers of interpretation through the development of new analysis methods and protocols (Cui et al., 2012).

Montague et al. (2002) identified a significant limitation within the field in which most studies only record the brain activity of one person at a time and fail to take into account any synergistic or antagonistic brain activity between multiple participants during social teaming. However, to address this limitation, a new experimental paradigm was developed using hyperscanning to study the underlying mechanisms of social interactions.

Hyperscanning is a technique that consists of recording brain activity from multiple subjects simultaneously while also simultaneously ensuring the temporal synchronization of these data, such that recorded signals can be compared between and across individuals (Montague et al., 2002). This technique was first applied within functional magnetic resonance imaging (fMRI) studies (Chiu et al., 2008; Kauppi, 2010; King‐Casas et al., 2005; Li et al., 2009; Montague et al., 2002; Saito et al., 2010; Schippers et al., 2010; Tomlin et al., 2006) and was later expanded to include studies utilizing functional near infra‐red spectroscopy (fNIRS) (Babiloni & Astolfi, 2014; Cui et al., 2012; Dommer et al., 2012; Funane et al., 2011; Liu et al., 2016; Scholkmann et al., 2013), magnetoencephalography (Ahn et al., 2018; Zhou et al., 2016), and recently to electroencephalography (EEG; Astolfi et al., 2010; Babiloni et al., 2006; Dikker et al., 2017; Dumas et al., 2011; Kinreich et al., 2017; Lindenberger et al., 2009; Mu et al., 2017; Pérez et al., 2017); for a tabular summary of findings from hyperscanning research utilizing each sensor technology, see Table 1, and for a more recent and detailed review of the hyperscanning literature, see (Czeszumski et al., 2020).

**TABLE 1:** List of the analyzed studies performed with hyperscanning methodologies (from the most recent to the oldest). A similar table gathering researches realized before 2013 can be found in Babiloni et al. (2014)

**Research area**	**Author(s)**	**Task**	**Subjects**	**Method**	**Results**
Turn‐taking verbal interaction	Ahn et al. (2018)	Number counting, speaking, and listening	10	2 x electroencephalography (EEG)/ magnetoencephalography(MEG)	Alpha and gamma oscillation with EEG and MEG in multiple regions followed by a phase synchronization between brain (phase lag index)
Brain‐to‐Brain synchrony	Dikker et al. (2017)	Group interactions in classroom	12	12 x EEG	Reduction of participant's alpha oscillation followed by an increase in alpha synchrony between participants. Higher interpersonal students had higher interbrain synchrony (IBS). External factors played a role in IBS (e.g., teaching style).
Neuroeconomy	Horat et al. (2017)	Ultimatum Game	20	2 x EEG	Modulation of ERPs (P2, FRN, LPC, and N2) depending on the role played and the outcome of the trial.
Social neuroscience	Kinreich et al. (2017)	Naturalistic social interaction	104	2 x EEG	IBS increased during social contact (eye contact, speech, etc.) and enhanced gamma band (parietal and temporal). The results were stronger for real couples when compared to the stranger's dyad
Social neuroscience	Mu et al. (2017)	Article reading and time counting	90	2 x EEG	IBS of gamma‐band oscillations is enhanced when people are under high threat, which comes with a lower dyadic interpersonal time lag (i.e., higher coordination)
Brain‐to‐brain synchrony	Pérez et al. (2017)	Naturalistic social interaction	30	2 x EEG	Inter‐brain synchronization was enhanced for delta, theta, alpha, and beta frequency band between dyads. The different frequency bands are affected differently depending on the condition (listener/speaker)
Environment perception and evaluation	Sciaraffa et al. (2017)	Flight Simulator–NASA MATB	10	2 x EEG	Significant variations in frontal theta and overall alpha‐band activity were found between participants, and they are associated with the mental workload. Mental workload is affected by the difficulty of the task. Higher workload begets less efficient coordination as participants focused on their respective tasks
Brain‐to‐brain synchrony	Szymanski et al. (2017)	Visual search task	52	2 x EEG	IBS enhancement in frontoparietal delta and theta band during collaboration when compared to individual conditions
Brain‐to‐brain synchrony	Sinha et al., (2016, 2017)	Pong game	24	2 x EEG	IBS significantly enhanced in alpha and beta band. Only cooperation elicited significant activation–not competition. Higher IBS during collaboration than competition
Neuro‐management	Labonté‐Lemoyne et al. (2016)	Role‐Play of different management style	42	2 x EEG	Alpha band in the right frontal region changes depending on the flow state of the participant. Flow induced more alpha activation. Players in a boredom state were significantly paired with high alpha partners
Neuro‐management	Toppi et al. (2016)	Flight simulator	12	2 x EEG	Significantly higher IBS between a trained pilot dyad compared to unskilled + skilled pilot dyad. Higher collaboration phase induced significant activation in theta and a trend toward higher alpha band activity. The leader (flight operator) showed significant solo activation in the frontal, parietal, and occipital areas during certain phases (take‐off and landing), suggesting higher engagement of resources during these periods
Imitation and perception	Zhou et al. (2016)	Imitation of hand movements	16	2 x MEG	Alpha and beta modulation in sensorimotor cortices. In the occipital region, beta modulation was stronger for the leader than the follower. The modulation is significantly caused by the role of the participant
Environment perception and evaluation	Delaherche et al. (2015)	Imitation of hand movements	5	2 x EEG	Significant difference in alpha, beta 1 and 2, and also gamma but only between imitation versus non‐imitation condition were found between participants
Imitation and perception	Konvalinka et al. (2014)	Synchronized finger tapping	18	2 x EEG	Asymmetric patterns of the frontal alpha‐suppression in each pair, during anticipation and execution task, such that only one member showed the frontal component. Analysis of the behavioral data showed that this distinction coincided with the leader–follower relationship in 8/9 pairs, with the leaders characterized by the stronger frontal alpha‐suppression. This suggests that leaders invest more resources in prospective planning and control

Hyperscanning studies utilizing EEG has been used to investigate collaborative team task‐orientated social interaction in several contexts, such as when playing music (Babiloni et al., 2012; Lindenberger et al., 2009), observing team dynamics and the cerebral processes involved while playing the “chicken's game” derived from game theory (Astolfi et al., 2012; Toppi et al., 2016). Moreover, hyperscanning has been used to investigate social decision‐making neuroeconomics such as when playing the ultimatum game (Horat et al., 2017; Yun et al., 2008) or in the trust game (King‐Casas et al., 2005). These studies show that hyperscanning is fast becoming a reliable and contributive technique for advancing social neuroscience knowledge.

The common theme linking this body of research is the hypothesis that collaboration is associated with frontal and prefrontal cortical synchronization, a phenomenon likened to a “tuning” effect between individuals. However, it remains uncertain whether collaboration is associated with an increase of IBS within specific frequency bands as the dynamics of inter‐individual frequency modulations has yet to be studied in detail (Astolfi et al., 2010; Dumas et al., 2010; Pérez et al., 2017; Sinha et al., 2017). Consequently, studies that refine and increase the depth of our understanding of brain dynamics and inter‐individual collaborative effort mechanisms will contribute to the further development of the field and related applications.

To contribute both to a refinement of methods and add depth to our understanding of the neurophysiological changes in activity associated with collaborative and competitive effort, we report methods and results from a study that seeks to replicate and extend the research performed by Cui et al. (2012). We further develop the experimental protocol using EEG in place of fNIRS to assess whether collaboration is associated with specific frequency modulations of neurophysiological activation. We seek to determine if collaboration is linked to an increase of IBS while performing a collaborative task. Furthermore, we aim to assess if the level of IBS is correlated with performance. We hypothesize that collaboration while performing a task will induce collaboration‐specific cortical activation and that this activation will be located predominantly in frontal cortical areas and expressed within the alpha frequency band. That IBS between participants will increase during collaboration and that increase in IBS will be correlated with performance.

## 2 METHODS

### 2.1 Subjects

Forty‐six (23 dyads) healthy participants (17 female), aged (μ = 26.48, σ = 4.04) were recruited on the basis of normal or corrected to normal vision and no history of neurological disorder to take part in the experiment. Dyads were randomly selected throughout the sample, and participants possessed no prior knowledge of each other prior to taking part in the experiment. Data from three dyads were redacted due to technical issues impacting data recording. Each participant was compensated $CAD 30 following the experiment. The study protocol was approved by the Institutional Review Board of our institution (#2015‐1533‐1529), and participants provided written informed consent prior to participation.

### 2.2 Experimental protocol

**FIGURE 1:**
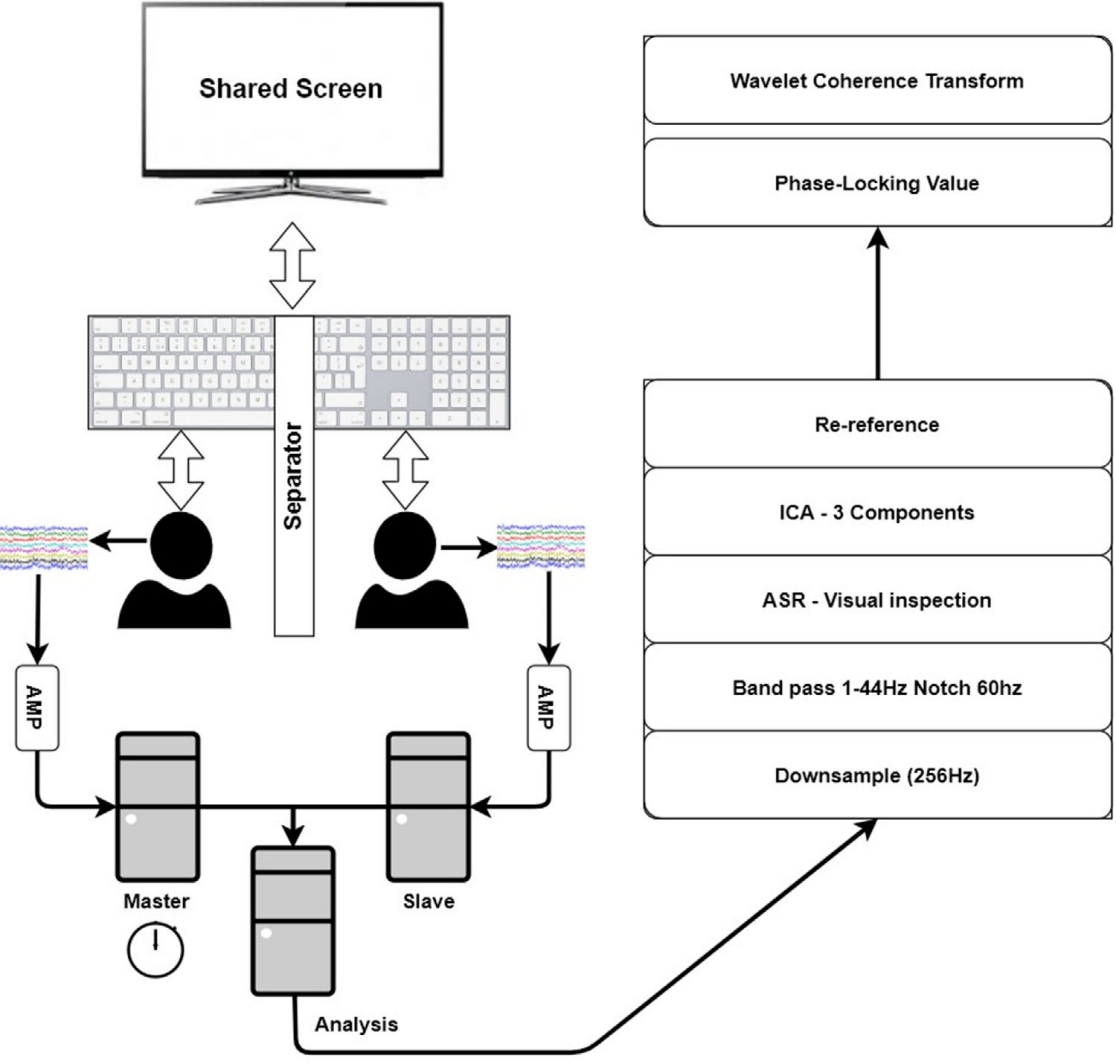
Diagrammatic representation of the experimental setup, depicting a dyad of participants performing tasks and outlining the signal processing and analysis methods. Participants are facing a shared screen with a splitter panel installed on the shared keyboard, preventing them from seeing each other hand movements

The protocol used for this study is an iteration and replication of the study performed by Cui et al. (2012). The experiment consists of a computer‐based game divided into four conditions (collaboration, competition, single 1 & 2) where both players from each dyad are seated side‐by‐side. The collaboration and competition conditions aim to investigate the potential cortical activation related to these specific interactions. The two single conditions act as controls to contrast each individual's normal levels of cortical activation related to the competition and collaboration conditions independently. Dyad participants play on the same computer screen using the same keyboard. A splitter panel is installed on the keyboard in order to prevent them from seeing each other's hands (see Figure 1). Participants are asked not to speak or move during testing; several breaks were offered to allow rest. The experimental tasks were divided into two distinct blocks of 30 trials each, separated with a 30 s resting period, such that each condition consists of three parts block1, rest, block2. The order of tasks performed within a block for each of the four conditions was randomized. For example, a dyad might complete the four tasks in the following order: single 2, competition, cooperation, single 1.

### 2.3 Cooperation

**FIGURE 2:**
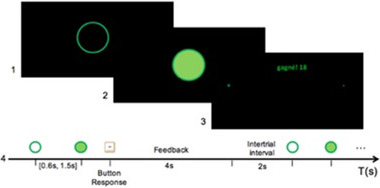
Task flow of the cooperation experiment. (a) The ready signal, (b) the “go” signal indicating participants are to press their assigned button, (c) feedback window for a given trial. The word “*gagné*” means “win” in French, and (d) time in seconds

For the cooperative condition, a hollow green circle is presented at the beginning of each trial (see Figure 2), and a 2‐s interval separates each trial. After a random delay of 0.6–1.5 s (uniformly distributed), the green circle was filled with a lighter green disk (“go" signal). Participants were instructed to press their respective response keys only after the “go” signal. The time between the “go” signal and the keypress was captured and stored as “response time” (RT). If the difference between the RT of the two participants was smaller than a threshold (Equation 1), both participants earned one point; otherwise, they both lost one point. Participants were instructed to maximize the number of points earned. The participant on the right (denoted as participant 1) was instructed to use the “2” key, and the participant on the left (denoted as participant 2) was instructed to use the “z” key. A feedback screen was displayed for 4 s at the end of each trial showing the total number of points accumulated. This screen also indicated feedback to the participants using a green symbol on the left and right edges of the computer screen highlighting which participant was the fastest (+) and slowest (–), a 2‐s inter‐trial interval separated each trial.

### 2.4 Competition

The same experimental timeline and settings were used for the competition condition. However, in this case, each participant within a dyad was instructed to respond faster than their counterpart at the “go” signal. For each trial, the participant who responded more quickly won a point, while the other kept the same amount of points. If a participant responded before the “go” signal, they lost a point. To reduce the effectiveness of anticipatory responses, the timing of the “go” signal onset was randomized (0.6–1.5 s; uniformly distributed) as in the cooperation condition. The feedback screen displayed the word “win!” on the winner's side and the word “lose!” on the loser's side for 1.5 s. Additionally, for consistency purposes, in the cooperation condition, the screen showed the (+) and (–) signs corresponding to which player was the fastest and the slowest and the cumulative score for each participant.

### 2.5 Single 1 and 2

For the control conditions, all experimental settings were identical to the competition task, except that in this case, only one participant was responding while the other passively observed the screen. As in previous conditions, the active participant was instructed to press their key as quickly as possible upon seeing the “go” signal, while the passive participant was instructed to observe the screen passively. No time limit was imposed upon the responder; if the responder pressed a wrong key on the keyboard, they lost a point. The feedback screen displayed responder points. At the end of the task run, the participation role activity was reversed to allow the current passive participant to become the active participant and complete the same task.

### 2.6 Data acquisition and preprocessing

We recorded continuous EEG signal using two 32‐electrode geodesic sensor net arrays and Netstation acquisition software and EGI amplifiers (Electrical Geodesics Inc). Impedance levels were measured as below 50 kΩ with a sampling rate of 500 Hz with no online processing performed. The experimental stimuli were presented using E‐Prime 2.0 software (Psychology Software Tools Inc.) on a 22‐inch LCD screen with a resolution of 1680 × 1050 pixels. Each EEG sensor net had a separate dedicated signal amplifier and acquisition computer.

To synchronize EEG signal acquisition within a dyad, one participant's signal amplifier and acquisition computer were designated as the master clock, with the other set as “slave” to the master clock. This process provided data synchronization within 2 ms (±1 ms). Experimental event markers were synchronized with these data and imported with the use of Noldus' Observer XT and Syncbox (Noldus Information Technology).

EEG raw data were transformed via conversion into a Matlab compatible file using BrainVision Analyzer 2.1.1 (Brain Products GmbH). EEG analysis was then performed using Matlab 2018a (Mathworks) and the EEGLab Toolbox 14.1.2b (Delorme & Makeig, 2004). The EEG signal was first downsampled to 256 Hz, and a bandpass filter of 1–40 Hz (60 Hz notch) was applied. Noisy channels or data segments were identified and marked using artifact subspace reconstruction (SD = 20) and visual inspection (C. Y. Chang et al., 2019; Plechawska‐Wojcik et al., 2018). Independent component analysis (ICA) using the ICA toolbox (Makeig et al., 2000) with extended infomax set to 1 was applied to the resulting signal data for all electrode sources to classify signal variance associated with vertical and horizontal eye‐blinks and heart rate (where applicable), with a maximum of 12 components. When identified, these components were corrected through manual inspection and automated action (Jung et al., 2000). An average of 2.05 components was removed (SD = 0.61).

All EEG signal was re‐referenced offline using the common linked mastoid average reference. An FFT was applied to the EEG data to split the signal into frequency bands, and then the theta (4–7 Hz), alpha (8–12 Hz), and beta (13–30 Hz) frequency bands were extracted; these data were then segmented into 3‐s epochs starting 1 s (–1–2 s) before stimulus presentation.

### 2.7 Data analysis

An analysis of wavelet coherence, also known as wavelet transform coherence (WTC), was conducted to assess the relationship between the EEG signals from both members of a dyad for each frequency band. WTC is an analysis method that measures the cross‐correlation between two time series as a function of frequency and time (Balconi & Vanutelli, 2017; Cui et al., 2012; Torrence & Compo, 1998). It can be considered as the local correlation between two time series (Grinsted et al., 2004). WTC is a useful analysis method that allows for local phase‐locked phenomena to be uncovered that are not be discernible using traditional time‐series analysis (Cui et al., 2012; Grinsted et al., 2004). This analysis method is widely applied in specific fields such as meteorology (Torrence & Compo, 1998) and seismology (Grinsted et al., 2004). However, according to Addison (2017), it remains an infrequent analysis method for neuroscience using EEG signal though precedent was set in both fMRI and NIRS (C. Chang & Glover, 2010; Cui et al., 2012) studies, which utilized a hyperscanning experimental protocol.

**FIGURE 3:**
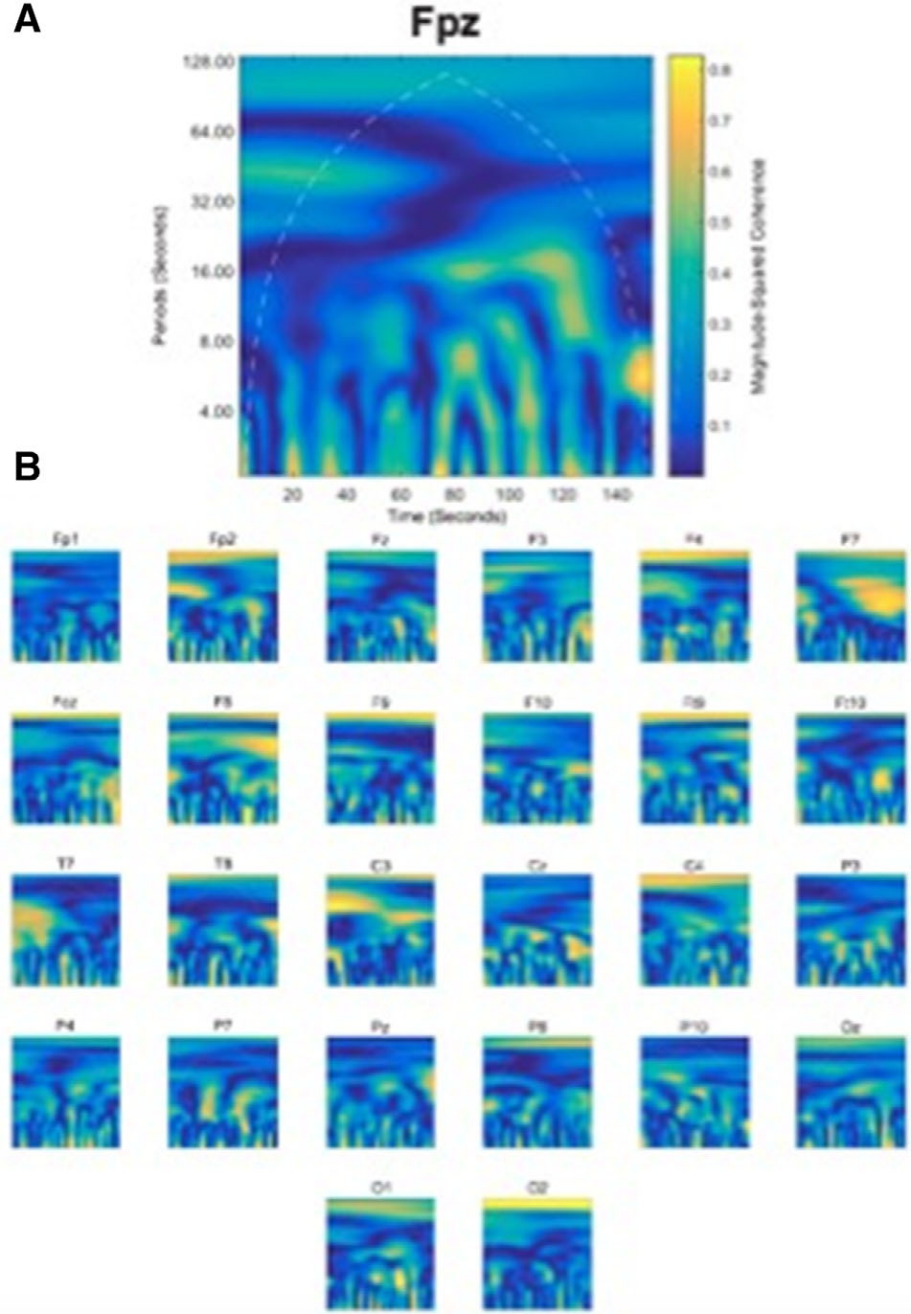
(a) Example coherence map showing dyad (#1) coherence synchrony in the alpha frequency band for channel Fpz, (b) alpha frequency band coherence synchrony for all electroencephalography channels for dyad (#1)

To perform the WTC analysis reported here, the Matlab Wavelet Toolbox (MathWorks Inc.) was utilized. For each channel from each dyad of participants in the cooperation experiment, we had frequency band time‐series data (e.g., theta, alpha, and beta in FPz from participant 1 and theta, alpha, and beta in FPz from participant 2). These time series were obtained by calculating an average frequency band power value for each frequency at every second of the recording. WTC analysis was performed on these data and generated both numerical output and a 2‐D coherence map (Figure 3). Within each task block, there are 30 trials. We aggregated three stimulus events to create a 9‐s fixed interval (3.2–12.8 s) in which we posited meaningful task–brain activity occurred, giving an average of 10 fixed time intervals. We then calculated an average coherence value for each frequency band during the two task blocks (block1, block2) of each experimental condition (collaboration–competition). The same procedure was then applied to data from the within condition resting period between each task block.

**FIGURE 4:**
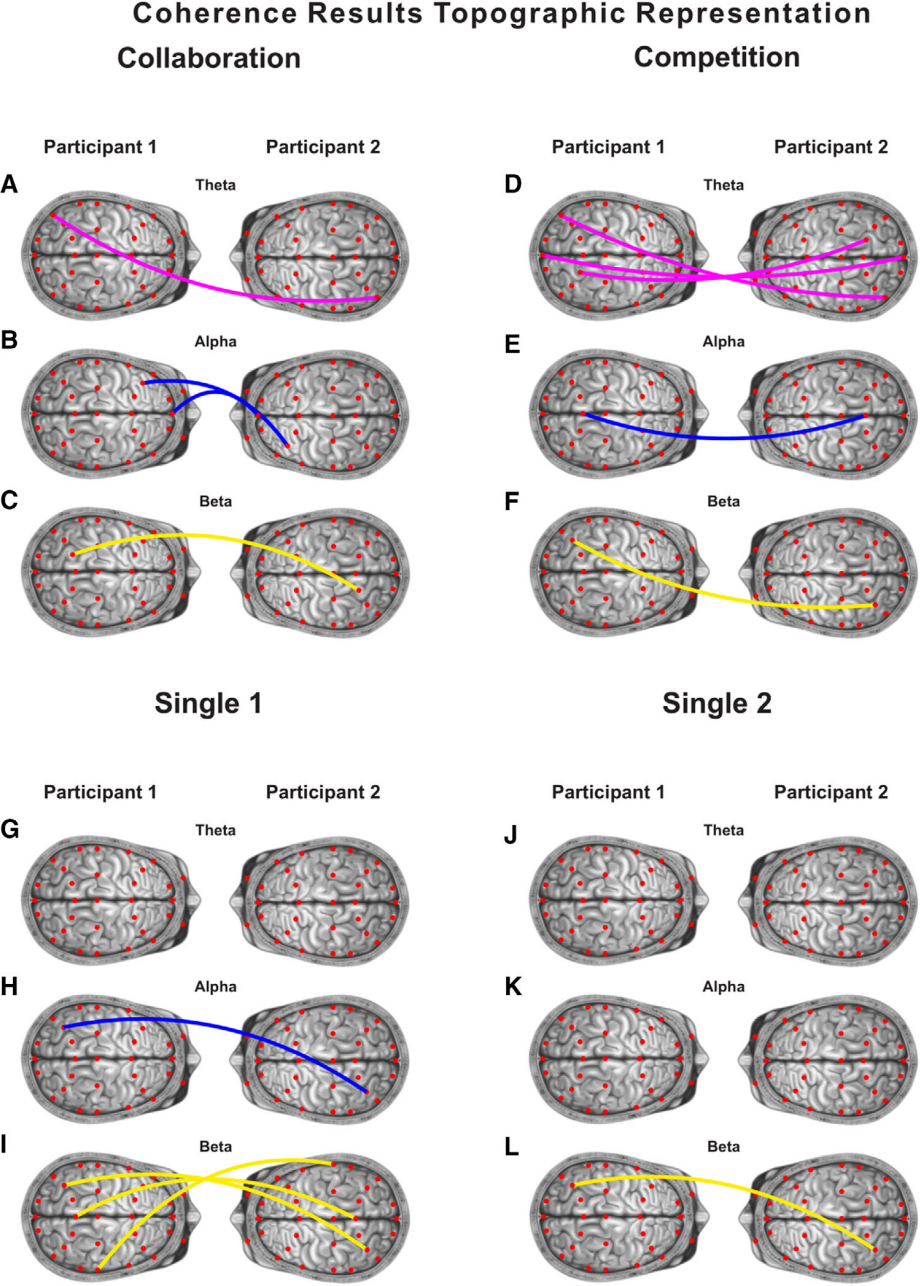
Topographic representation of coherence results. Illustrative connections represent a significant level of coherence between the two participants (*p* < .05). Each color is associated with a frequency band: purple–theta, blue–alpha, yellow–beta

Additionally, we calculated IBS as a “coherence increase” index defined as the average coherence value for the two task blocks minus the average coherence value in the task rest period (Equation 2). To indicate positive synchrony, we set a threshold value of one half, above which increased synchrony was shown to have been achieved (Cui et al., 2012). Once calculated for each channel and frequency band, coherence increase values were converted to Fisher z‐statistics (C. Chang & Glover, 2010; Cui et al., 2012). Finally, a one‐sample *t‐*test of “coherence increase” was performed across all participant dyads. Significant results (*p* < .05) are represented in Figure 4 as indicating dyadic coherence synchrony.

(1) $$IBS\;=\frac{1}{2}\;\left(IBS_{block1}+IBS_{block2}\right)-IBS_{Rest}$$

where inter‐brain synchrony index (*IBS*) *is the mean coherence value–IBS_block_*_1_ and *IBS_block_*_2_ are the coherence values for task block 1 and 2*. IBS_Rest_ *is the coherence value during the resting period.

In addition to WTC analysis, single‐subject analyses were performed to compare frequency band modulation across every condition in order to contrast the WTC results. Thus, all epochs of frequency band power from a given condition were averaged, and analysis of variance (ANOVA) was performed with frequency band power as a within‐subject factor across all of the four conditions. As a post hoc test, paired‐sample *t*‐tests were performed on significant ANOVAs. In order to avoid either type I or type II error, we fixed a significance level of 5% determined with a 10,000‐iteration permutation test, for both ANOVA and paired‐sample *t*‐test.

(2) $$S\;=\frac{1}{8}\;\left(R1+R2\right)$$

Equation (2) indicates response times threshold. *S* is the threshold–*R1* and *R2* are the response times of the participants 1 and 2. The parameter 1/8 was chosen to ensure the task achieved a reasonable level of difficulty.

Finally, reaction times were defined as the duration in milliseconds between stimulus presentation and participant button response. For each condition, a mean reaction time value was calculated. ANOVA was performed with reaction time as a within‐subject factor across all conditions. Note that the single 1 and single 2 conditions, where one participant observes the other performing the task, are not included in these behavioral analyses. Task performance was calculated as the number of points accumulated and converted into a percentage for each condition. Correlation analyses were performed in order to assess the relationship between performance and both “coherence increase” and single subject cortical activation.

*Phase‐locking value (PLV)*

To explore a different and potentially complementary IBS measure and how this compares with wavelet coherence, the EEG data were analyzed to derive the PLV to detect synchronicity between each dyad's brain recording (Dumas et al., 2010). EEG data were first filtered to the desired frequency band (theta, alpha, and beta) using a finite impulse response filter (Lachaux et al., 1999; Namburi, 2011). Data were then transformed using Hilbert methods for specific bands: theta (4–8 Hz), alpha (8–13 Hz), and beta (13–30 Hz). Finally, the PLV was extracted/calculated using a Matlab function (Namburi, 2011). Thus, for a given dyad, these data consisted of a vector of 768 samples of PLV per condition and electrode. Following Namburi's (2011) guidelines, the first and last 100 samples of each stimulus epoch were discarded to remove edge artifacts due to the PLV transform, leaving 568 samples for a given dyad condition and electrode pair.

To determine whether the PLV were significantly different, we then averaged all PLV for each dyad, per stimulus epoch for a given condition and electrode. We then performed a repeated‐measures ANOVA (*p*‐values Bonferroni‐corrected for multiple comparisons) with a three‐by‐four design using frequency (3) and task condition (4) as fixed factors for each target electrode. Electrode targets were derived from the significant activity reported from the WTC analysis. Only significant values with the Greenhouse–Geisser correction applied were reported (Greenhouse & Geisser, 1959).

## 3 RESULTS

Our goal using frequency band activity in sensor space as a proxy to infer increased activation was to determine if collaboration was associated with specific cortical activation by assessing the cortical activation synchrony of multiple dyads using wavelet coherence and PLV.

### 3.1 Coherence results

Shown in Figure 4 is a topographical representation of the coherence results. In this figure, connections represent false detection rate (FDR)‐corrected significant synchrony between dyad participants for each condition. All coherence results were produced via ANOVA (FDR‐corrected) analysis of the fixed period interval (3.2–12.8 s) discussed in Section 3. This period includes the trial period (∼7 s), inferring that any reported coherence synchrony increase during this interval is task‐related.

In the collaboration condition higher coherence levels were reported in Fpz (*t*(18) = –2.077, *p* = .042) and F7 (*t*(17) = 2.239, *p* = .039) in the alpha frequency band. Similar increases in coherence in the alpha frequency band were also observed in the competition and single 1 conditions at Pz (*t*(15) = –2.223, *p* = .042) and P7 (*t*(17) = 3.476, *p* = .003) respectively. In this analysis, both the collaboration and competition conditions showed significantly increased theta frequency band coherence synchrony in both occipital and parietal electrodes. More precisely, for the competition condition, higher coherence synchrony was detected at Oz (*t*(17) = 2.813, *p* = .012) and in both the left P9 (*t*(14) = 4.737, *p* < .001) and right P4 (*t*(18) = –2.225, *p* = .039) parietal electrodes, while those in the collaboration condition demonstrated higher coherence synchrony at P9 only (*t*(14) = –2.126, *p* = .042). This compares favorably against the single conditions that did not show a similar theta frequency band coherence synchrony increase (*p* > .05). However, enhanced levels of coherence synchrony were found in parietal electrodes in the beta frequency band across all conditions (P3_collaboration_, *t*(18) = 2.388, *p* = .028; P7_competition_, *t*(17) = 3.476, *p* = .003; T8_single_1_, *t*(16) = –2.269, *p* = .037; P7_single_1_, *t*(17) = 2.345, *p* = .031; Pz_single_1_, *t*(15) = 3.878, *p* = .002; P7_single_2_, *t*(17) = 2.730, *p* = .014). An illustrative coherence map from a dyad of subjects can be seen in Figure 3.

### 3.2 Single‐subject results

The coherence synchrony increase observed in several conditions and frequency bands present interesting results. However, for completeness, these results require comparison with single‐subject trials to ascertain if any significant social‐condition‐specific effects remain. To determine this, we compared frequency band power in the alpha, beta, and theta bands across conditions through ANOVA and paired *t*‐test post hoc testing.

The results from the ANOVA analysis reported significant differences in all frequency bands for all conditions. However, only the competition condition was determined to be significantly different from all other conditions after post hoc testing (*p* < .05, FDR‐corrected). First, differences were observed in the theta frequency band in frontal, frontal left, and left temporal electrodes (*p* < .05). Moreover, post hoc tests confirmed that social conditions were associated with higher theta power in frontal (F7, Fz) and temporal (T8) regions (*p* < .05) when compared to single conditions. Furthermore, post hoc tests also revealed that competition was different from all other conditions in the frontocentral FCz (*p* < .05). Second, significant differences were found in the alpha frequency band in frontal and parietal electrodes (*p* < .05). Post hoc testing confirmed that social conditions are associated with activity in both the frontal left (F3) and frontal right (F8) electrodes, with significant activity also reported in the parietal electrodes (Pz, P7, P10; *p* < .05). Third, significant differences in beta frequency band were found in frontal/frontal right, temporal left, and right parietal electrodes. Post hoc testing confirmed differences between social conditions compared to non‐social single conditions in frontal (Fz, F10), temporal left (T7), and right parietal (P10; *p* < .05).

### 3.3 PLV results

To analyze PLV data, we utilized the significant results reported from the wavelet coherence analysis to target specific electrodes to determine if significant frequency activity remained consistent across different measures of synchronous activity. The aim was to determine if the same significant activity remained consistent between these two different and potentially complementary forms of analysis.

We utilized a three‐by‐four repeated measures ANOVA design to test the main effect of frequency (3) variation within task conditions (4). Where significant effects were found, additional pairwise comparisons (Bonferroni‐corrected) were performed.

As per the WTC analysis, the frontal Fpz, F7, F3, F8, Fz, F10, Fp1, Fp2; parietal Pz, P7, P4, P9, P3; temporal T8, T7; and occipital Oz electrodes were selected as targets for testing. While not strictly necessary for this form of analysis, for completeness, we detail any significant reported effects, as these may potentially increase our understanding of overall frequency band activity during social collaborative and competitive activities when compared to single active and single passive activities.

#### 3.3.1 Frontal

For electrode Fpz, the test of within‐subjects effects reported a significant variation in frequency (*F*(2,11.670) *p* = .000). However, no significant main effect was reported for task condition or interaction between frequency variation within task condition. Pairwise comparisons showed that the significant effect of frequency was predominantly in the theta band, compared to beta (*p* = .001). In the cases of Fp1 and Fp2, the test of within‐subjects reported a significant variation in frequency in both cases Fp1 (*F*(2,6.603) *p* = .009), Fp2 (*F*(2,3.685) *p* = .039). Pairwise comparisons showed that for Fp1, the frequency effect was in the beta frequency band when compared to theta (*p* = .008). However, Fp1 comparisons failed to report any significant variance. In both cases, no significant main effect within task condition or interaction between frequency variation within task condition was reported.

The tests for electrode F3 reported a significant variation in frequency (*F*(2,7.340) *p* = .003). However, no significant main effect for task condition or interaction between frequency variation within task condition was reported. Pairwise comparisons showed that the significant effect of frequency was predominantly in the beta frequency band, compared to theta (*p* = .006). Similarly, the tests performed for electrode Fz reported a significant variation in frequency (*F*(2, 9.284) *p* = .001). However, no significant effect of task condition or frequency variance within task condition was reported. Pairwise comparisons showed the frequency effect to be predominantly beta when compared with theta (*p* = .001) activity. No significant effects were reported for electrodes F7 and F8 in any frequency band or task condition.

Additionally, a significant effect of frequency was reported for electrode F10 (*F*(2,4.680) *p* = .016) and frequency within task condition (*F*(6,2.755) *p* = .034). Pairwise comparisons showed the variance in frequency was principally in the beta frequency band when compared with theta (*p* = .017). However, the significant interaction of frequency within task condition failed to reach significance in any task group comparisons.

#### 3.3.2 Parietal

Moving to parietal electrodes, the test of within‐subjects for electrode P3 reported no significant variation within the frequency, task condition, or frequency within task condition. However, the test of within‐subjects for electrode P4 reported a significant variance in frequency (*F*(2,19.736) *p* = .000), and frequency by task condition (*F*(6, 3.315) *p* = .024). Pairwise comparisons showed that this effect of frequency variance occurs in the theta band during the collaborative task condition when compared with the competition (*p* = .041) and also when compared with the single active condition (*p* = .004).

The tests for electrode Pz reported a significant variation in frequency (*F*(2, 9.597) *p* = .001). However, no significant effect of task condition or frequency variance within task condition was reported. Pairwise comparisons showed the frequency effect to be predominantly in the theta band when compared with beta (*p* = .001). Similarly, with P7 a significant variation in frequency was reported (*F*(2,22.237) *p* = .000). However, no significant main effect of task condition or interaction between frequency within condition was reported. Pairwise comparisons showed that the variance in frequency was predominantly for greater theta band activity when compared with alpha (*p* = .000) and beta (*p* = .000).

For electrode P9 a significant variation in frequency was reported (*F*(2, 9,772) *p* = .003), and pairwise comparisons showed this variance to be predominantly in the theta frequency band when compared with alpha (*p* = .012) and beta (*p* = .022). However, this activity failed to reach significance for frequency within task or task condition. Similarly, for electrode P10, a significant variation in frequency was reported (*F*(2, 10.787) *p* = .002), and pairwise comparisons showed this variance to be predominantly in the beta frequency band when compared with theta (*p* = .002). However, this activity failed to reach significance for task condition or frequency within task condition.

#### 3.3.3 Temporal

In the case of electrodes T7 and T8, the test of within‐subjects reported a significant variation in frequency for T7 (*F*(2,5.360) *p* = .014). However, no significant activity was reported in pairwise comparisons in any frequency but rather a trend toward higher beta frequency activity (*p* = .053), compared with theta activity. In both cases, no significant main effect within task condition or interaction between frequency band variation within task condition was reported.

#### 3.3.4 Occipital

The test of within‐subject effects for electrode Oz reported a significant main effect of frequency (*F*(2,19.308) *p* = .000) and task condition (*F*(3,3.215) *p* = .042). Pairwise comparisons showed that the frequency variance was predominantly for greater theta activity, compared to alpha (*p* = .18) and beta (*p* = .00). Further comparisons showed that the significant theta activity was present in the collaborative task condition when compared to the single active task (*p* = .013) and trending toward significance, compared with the single passive task (*p* = .055). This significant activity remained when comparing frequency within task condition, comparing collaboration with single active task conditions (*p* = .015). However, the trend toward significance comparing collaboration with the single passive task condition disappeared.

### 3.4 Behavioral results

**FIGURE 5:**
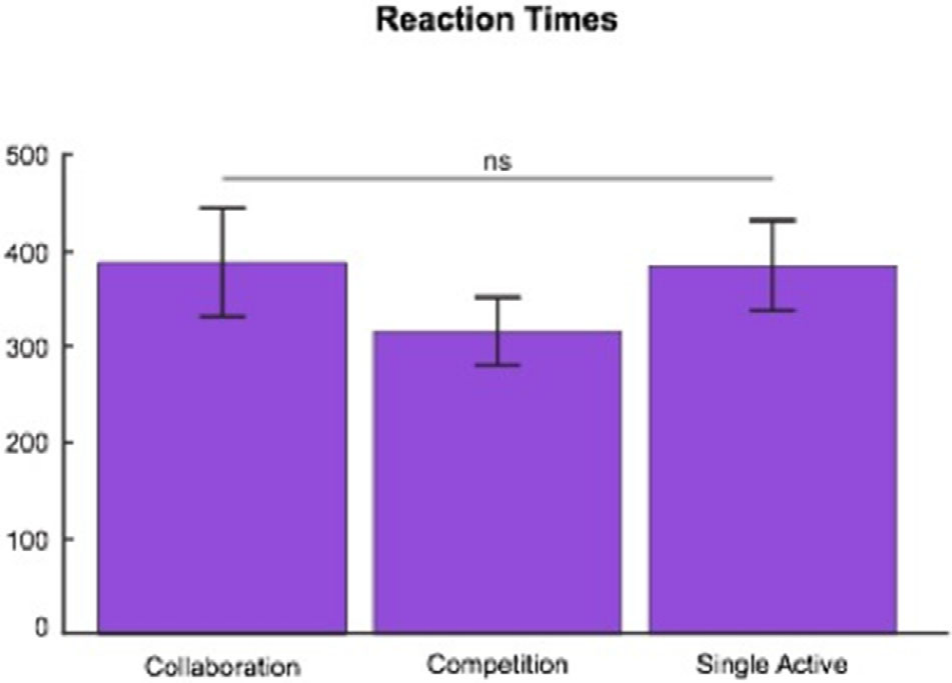
Mean and variance in reaction times across conditions

We compared the mean reaction times between collaboration, competition, and single‐active conditions. We did not find any significant differences across these conditions (*p* = .056; see Figure 5).

Concerning performance, correlations with reports of increased coherence synchrony values in any channels were not significant (*p* > .05) for any of the conditions. However, correlations between performance and single subject activation showed significant results in collaboration. In fact, activity in the alpha frequency band in F4 is negatively correlated with performance (*r* = –0.338, *n* = 40, *p* < .05). Although, performance is also correlated with beta frequency band in Fz (r = –0.350, *n* = 40, *p* < .05), Fpz (*r* = –0.484, *n* = 35, *p* < .01), Fp1 (*r* = –0.357, *n* = 35, *p* < .05), and Fp2 (*r* = –0.384, *n* = 35, *p* < .05). Other conditions were not correlated with performance.

## 4 DISCUSSION

This study aimed to replicate the work of Cui et al. (2012) by adapting the reported hyperscanning experimental procedure using synchronous EEG recordings taken from dyad participants, to assess the level of coherence synchrony between EEG frequency bands during collaborative and competitive social tasks. We utilized both wavelet coherence transform and PLV as measures from which to perform our analysis. In this regard, it appears that for this study, WTC is potentially more sensitive to frequency variance than PLV for instances involving IBS during the task conditions. Moreover, while PLV was less sensitive in some of these instances, it did capture significant gross frequency activity at the same locations as the WTC providing some convergent validity to our results.

### 4.1 Hypothesis testing

In terms of our initial hypothesis, that collaboration while performing a task will induce collaboration‐specific cortical activation. The EEG data analysis results provide some evidence to support this hypothesis, indicating an increase in coherence synchrony in the prefrontal cortex between dyad members in the alpha frequency band during the collaborative task. Therefore, we postulate that tasks induce collaboration while performing collaboration‐specific cortical activation focused in frontal cortical areas within the alpha frequency band and that coherence synchrony occurs between participants during collaboration. There is support for this assertion provided by the identification of phase‐locked alpha (9—14 Hz) in the centro‐parietal region during coordinated hand movements (Dumas et al., 2010) and imitated finger‐pointing (Naeem et al., 2012). Furthermore, the parietal cortex has been implicated in the flexible representation of task‐reward and later switching to task processing (Wisniewski et al., 2015). This may help explain our observed parietal beta frequency activity across all conditions, notably competition, in which a constant reevaluation of actions associated with perceptions of self and others is required to complete the tasks, which switches to a purely “self” evaluation with associated reward and cognitive load balancing mechanisms for competitive task completion. However, this increase in coherence synchrony between participants does not equate to increased task performance in terms of reaction speeds. Furthermore, it is our opinion that this lack of performance increase is not generalizable to other forms of collaborative task but rather an artifact of this specific synthetic task.

Moreover, it was observed that collaborative effort appears to induce phase‐locked coherence synchrony of cortical activity in the theta frequency band. The occurrence of theta band activity during collaborative efforts is an interesting finding and one that has been previously observed (Pérez et al., 2017; Sciaraffa et al., 2017; Szymanski et al., 2017; Toppi et al., 2016) yet remains largely unexplained. There is some evidence to suggest that the processing of stimuli is controlled through top‐down processing mechanisms that strongly influence the intrinsic dynamics of thalamocortical networks to create predictions concerning forthcoming sensory stimulus events constantly. It is suggested that these predictions may be embodied in the temporal structure of stimulus‐evoked and ongoing activity and that synchronous oscillations are of particular importance in this process (Engel et al., 2001).

### 4.2 Social alignment feedback loop as a mean to interpret coherence synchrony

Interpreting the increase in theta activity reported from our analysis in the context of coherent interpersonal interaction during an active task, we speculate that the observed increase in theta activity during the cooperative task could be related to the recruitment and synchronization of the mirror neuron (Iacoboni et al., 2005) and predictive coding systems (Clark, 2013) during cooperative task completion, serving to aid in the synchronization of explicit motor activity between both active agents. That is, when performing a cooperative task, executive control and synchronized motor activity may be facilitated through the mirror neuron system as a part of a feedback system mediated by perceived success or failure. Over time, this network‐specific feedback system could potentially allow for synchrony of brain and motor activities between cooperating human agents while performing a cooperative task as one of multiple elements within an intra‐interpersonal gestalt of top‐down processing. Shamay‐Tsoory et al. (2019) recently proposed a three‐component social alignment feedback loop consisting of three systems: one system to react to alignment and another system that reacts to misalignment third, which determines misalignment and realigns behavior at the point of perceived alignment. This social alignment feedback loop appears to provide a framework in which theta oscillations, mirror neuron, and predictive coding network activity and other high‐level factors such as motivational, empathetic, and cognitive energetical processes integrate with “ToM” (Gallagher & Frith, 2003) and top‐down processing to give rise to IBS during cooperative tasks.

Indeed, theta frequency oscillations have been shown to carry stimulus‐specific information to the visual cortex in the case of focused attention and are related to successful working memory performance (Ekstrom et al., 2005). Moreover, there is evidence that these oscillations have far‐reaching effects that branch out from the hippocampus to distant cortical areas (Caplan et al., 2003). Therefore, it may be fair to posit that IBS is facilitated by theta oscillations in a social context as each agent within a cooperative social task consciously or unconsciously attempts to synchronize behavior in order to complete the task. Thus, using the social alignment feedback loop as a framework to explain IBS, a recognition system activates to identify that cooperative synchronous activity that is required to complete a task. This then leads to mirror neuron and predictive coding network activation, and theta oscillations emerge to synchronize intra‐ and inter‐brain activity and task behaviors to provide a degree of behavioral mimicry potentiated by social dynamics mediated through higher‐level factors (ToM).

Alternatively, if we interpret our results in terms of similar task activity, this observed increase in theta activity in parietal and occipital cortices could be due to participants performing very similar actions to complete the task potentially enhanced by proximity in group settings. This explanation has been explored in studies involving listening to music (Abrams et al., 2013) and watching movies (Nummenmaa et al., 2012); in these studies, the emotional response was posited as the interbrain synchronizing factor. Another study investigating the synchronization of human‐machine speech rhythms (Kawasaki et al., 2013) posited the possibility that motor movement similarity could explain the theta‐alpha IBS. In these social context studies, participants performed the same tasks without any form of interpersonal interaction. However, IBS was observed after analysis, leading to a conclusion that similar tasks involve similar neurophysiological solutions, thus giving rise to the appearance of synchronous activity when data is analyzed post hoc.

In the current study, participants are in the same room performing the cooperative task simultaneously. Given this context, we would posit that the observed increase in theta activity emerges as part of cooperative interaction as opposed merely to a property of a shared task. However, a shared task effect cannot be ruled out, and future work with refined experimental design may highlight this effect.

As a replication study, the results reported in this manuscript are well aligned with those of Cui et al. (2012) concerning the increase in dyadic coherence synchrony taking place in the frontal cortex. However, our results provide additional insight in which we observed significant beta frequency parietal and occipital coherence synchrony in every condition. Furthermore, this multi‐frequency oscillatory dynamic across the cortical hierarchy has been recently observed (Lundqvist et al., 2020), indicating that identifying specific frequency band synchrony as evidence of dyadic task‐related adaptation may not be as strong as capturing a full‐spectrum heuristic of oscillatory and inferred neural activity. In light of our results, we can infer that coherence synchrony is not inherently coupled to a collaborative effort but rather that collaboration requires the recruitment of the prefrontal cortical areas, which is expressed as an observable increase in alpha‐band activity. However, contrary to the work of Cui et al. (2012), we did not find any significant difference in reaction times during collaboration but instead observed a similar trend regarding somewhat faster reaction times in the competition condition. Similarly, we did not find any correlation between inter‐brain synchrony and performance, leading to an interpretation of the results that favors the null hypothesis *that increases in IBS will not be correlated with performance* in this specific instance.

From a single subject perspective, we observed a condition‐related modulation of frequency band activity. We interpret these results such that task‐related social interaction can be associated with activation of specific brain regions, that is, prefrontal regions and higher frequency band power when compared to either passive or active single condition tasks. Such regions are consistently activated across different specific paradigms and experiments (Babiloni & Astolfi, 2014), not only in the isolated brain of a given participant but also when considering the coherence synchrony of both signals from a dyad of participants (Babiloni & Astolfi, 2014; Babiloni et al., 2007; Dikker et al., 2017). The presence of the activation of the prefrontal cortices in almost all of the research studies in the field strongly suggests that activity in the associative cortex contributes to social interaction (Babiloni & Astolfi, 2014; Cui et al., 2012; Toppi et al., 2015). Therefore, our findings add further evidence to strengthen the hypothesis that increased coherence synchrony during collaborative tasks and social interactions rely on the dynamic activation of frontal, temporal, and parietal cortical regions.

When taken as a whole, our analysis results provide additional, if tentative, evidence to confirm the role given to prefrontal regions in relation to social behavior. More precisely, the superior frontal cortex is considered by several researchers in the field as playing a central role in the ToM (Cui et al., 2012; Ritter et al., 2011). A pragmatic investigation involving certain populations such as individuals with autistic syndrome disorder (ASD) may further research in this area, through the comparison of brain activity of those from the ASD population with those classified as “neurotypical” under collaborative or competitive task conditions, considering that ASD has been associated with deficits in ToM (Baron‐Cohen, 2006).

## 5 LIMITATIONS AND FUTURE WORK

There are a number of potential research approaches that may add valuable data to the field. First, by investigating the dynamics of coherence synchrony increases with regard to the familiarity of dyad members to include the nature of their relationship (e.g. professional vs. intimate), it may be interesting to question whether higher familiarity is associated with higher coherence synchrony during a collaborative task. A second approach may investigate the reported correlation between performance and coherence synchrony increase through task difficulty or feedback modulation. For example, it would be possible to give predetermined feedback (false information) after each trial to dyad participants and observe how this affects IBS. It would then be possible to determine how feedback and perceived performance modulates a participant's brain activity. Third, the research reported here could be replicated as a technology‐mediated protocol, where participants are not located side‐by‐side locally but rather separated by locale. The goal would be to assess the importance of physical presence to social interaction and increases in coherence synchrony during task performance. Last, a more ecological task (e.g., a cooperative video game) could be used to investigate the relationship between brain activity and social interaction. Other than flight simulation studies (Astolfi et al., 2012; Toppi et al., 2016), few experimental studies utilize ecologically valid tasks and environments. However, some early work has been reported using a cooperative video game (Labonte‐Lemoyne et al., 2016), yet there remains a vast knowledge gap in our understanding regarding cooperative and competitive social interactions. Finally, considering the growing interest in the industry for new technologies that optimize team cooperation and efficiency, methods such as wavelet coherence transform and hyperscanning may become important tools to develop these technologies.

## 6 CONCLUSION

In summary, our results suggest that social interaction during collaborative and competitive tasks can be associated with brain activity in specific cortical regions, namely, the prefrontal and parietal regions expressed as higher frequency band power, compared to single condition tasks, either passive or active. Our results consolidate previous research findings that are converging toward the key role of the alpha and theta frequency band activity within the frontal and prefrontal cortices (Babiloni & Astolfi, 2014) and raise questions concerning the unclear role of the beta frequency band during collaborative or competitive tasks. From a methodological point of view, our study illustrates that wavelet coherence analysis applied to EEG hyperscanning data offers a powerful tool to study social cognition in a variety of contexts and populations. Not only does it contribute to increasing the depth of our understanding of the underlying brain dynamics and mechanisms of social interactions, but it also opens a completely new field of research‐oriented toward industry and the development of technologies focused on increasing team efficiency.

## CONFLICT OF INTEREST

The authors assert that they have no competing interests.

### TRANSPARENT PEER REVIEW

The peer review history for this article is available at https://publons.com/publon/10.1002/brb3.2270

## Data Availability

The data that support the findings of this study are available on request from the corresponding author. The data are not publicly available due to restrictions from the institutional review board (IRB) of our institution, as they contain information that could compromise the privacy of research participants.
